# Wound Infections from Taiwan Cobra (*Naja atra*) Bites: Determining Bacteriology, Antibiotic Susceptibility, and the Use of Antibiotics-A Cobra BITE Study

**DOI:** 10.3390/toxins13030183

**Published:** 2021-03-02

**Authors:** Heng Yeh, Shi-Ying Gao, Chih-Chuan Lin

**Affiliations:** 1 Department of Emergency Medicine, Lin-Kou Medical Center, Chang Gung Memorial Hospital, Taoyuan 33305, Taiwan; tsubame800329@gmail.com (H.Y.); s78092359@cgmh.org.tw (S.-Y.G.); 2 School of Medicine, College of Medicine, Chang Gung University, Taoyuan 33302, Taiwan

**Keywords:** wound infections, snakebites, Taiwan cobra, *Naja atra*

## Abstract

Wound necrosis and secondary infection are common complications after *Naja atra* bites. Clinical tools to evaluate the infection risk after Taiwan cobra bites are lacking. In this Cobra BITE study, we investigated the prevalence of wound infection, bacteriology, and corresponding antibiotic usage in patients presenting with Taiwan cobra snakebites. Patients with wound infection lacking tissue necrosis were included in developing Cobra BITE score utilizing univariate and multiple logistic regression, as patients with wound necrosis require antibiotics for infection treatment. 8,295,497 emergency department visits occurred in the span of this study, with 195 of those patients being diagnosed as having cobra bites. Of these patients, 23 had wound necrosis, and 30 had wound infection, resulting in a wound infection rate of 27.2% (53/195). *Enterococcus faecalis* and *Morganella morganii* were the main bacteria identified in the culture report regardless of whether patients’ wounds had necrosis. As per our Cobra BITE score, the three factors predicting secondary wound infection after cobra bites are hospital admission, a white blood cell count (in 103/µL) × by neu-trophil-lymphocyte ratio value of ≥114.23, and the use of antivenin medication. The area under the receiver operating characteristic curve for the Cobra BITE score system was 0.88; ideal sensitivity and specificity were 0.89 and 0.76. This scoring system enables the assessment of wound infections after *N. atra* bites, and it could be modified and improved in the future for other *Naja* spp. bites.

## 1. Introduction

**Figure 1:**
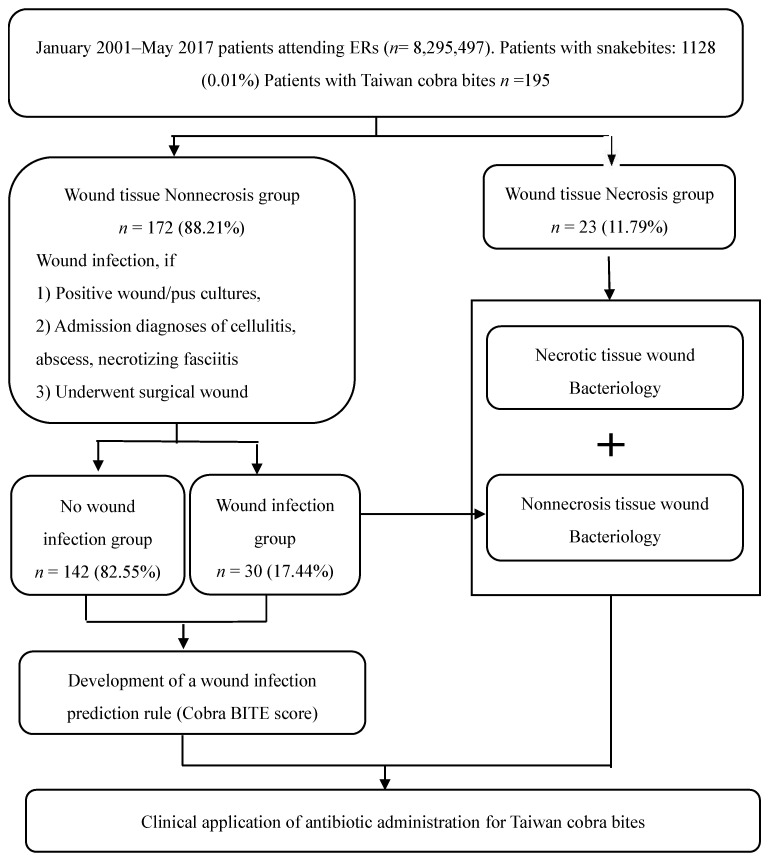
Study flow diagram for Cobra BITE score. The enrolled cobra bites patients were divided into wound tissue necrosis and nonnecrosis group. Patients were defined as having wound necrosis if they met the criteria of positive wound/pus culture or admission diagnoses of cellulitis, abscess, and necrotizing fasciitis. The nonnecrosis wound infection group was compared with the no wound infection group to develop the Cobra BITE score. The bacteriology of necrosis wound and nonnecrosis wound infection groups was employed to provide the clinical application of antibiotic administration for Taiwan cobra bites.

Taiwan, a subtropical island, has many types of snakes; of these, six species of venomous snakes are considered clinically important: *Naja atra* (Taiwan cobra or Chinese cobra), *Bungarus multicinctus*, *Protobothrops mucrosquamatus* (Taiwan habu), *Trimeresurus stejnegeri* (green bamboo viper)*, Deinagkistrodon acutus* (hundred pacer viper)*,* and *Daboia siamensis* (eastern Russell’s viper). Among these, *N. atra* are responsible for approximately 20% of venomous snakebites in Taiwan [1]. While other species of cobra cause neurological complications, Taiwan cobra bites do not; however, they cause wound tissue damage/necrosis due to the actions of cytotoxins in the venom, and confer a high risk of wound infection [2,3,4]. To our knowledge, there are no clinical parameters that can predict or assesses the clinical severity of the cobra bites patients. Although some studies have investigated the bacteriology of cobra bite wounds [5,6], guidelines for differentiating patients based on whether they have wound infections are lacking. Wound necrosis is a common complication associated with cobra bites [2], and affected patients may be treated with antibiotics. Knowledge on whether nonnecrotic cobra wounds are infected is important. Furthermore, to provide appropriate antibiotics to patients with wound infections, wound bacteriology must be investigated. This study investigated the wound bacteriology of necrosis wounds and infected wounds without necrosis to develop a clinically useful wound infection prediction rule (Cobra Bites/Bacteriology of Infections in Taiwanese snake Envenomation (Cobra BITE) study) to guide antibiotic use (Figure 1).

## 2. Results

### 2.1. Patient Characteristics

In total, data on 195 patients with cobra bites were retrieved from data on 8,295,497 emergency department (ED) visits from January 2001 to May 2017. The Chang Gung Research Database (CGRD) contains data on 1128 patients with snakebites (0.01% of total ED visits); men accounted for the majority of this study population (*n* = 144, 73.8%; mean age: 49.97 ± 17.42 years). Generally, two vials (median) of freeze-dried neurotoxic antivenin (interquartile range: 1–4) were administered to each patient, suggesting that these patients were mostly mildly to moderately envenomed. A total of 74 patients (37.9%) required hospital admission.

Of these 195 patients, 53 (27.2%) developed wound infections. Among them, 23 and 30 patients had wound tissue necrosis and nonnecrosis but wound infections. These 53 patients with wound infections received larger antivenin doses (median (interquartile range) = 2 (1–3) vs 2 (1–4), *p* = 0.002), had more admissions (*n* = 47 vs 27, *p* < 0.0001), and had longer hospital stays (3.07 ± 2.67 vs 16.09 ± 13.99, *p* < 0.0001) than patients without infected wounds, implying that their clinical conditions were severe. Patients with Taiwan cobra bites lacked neurological complications but had the most severely infected wounds, and they underwent the most serious envenomation and surgical procedures, such as debridement, fasciotomy, and graft. Of the 195 patients with cobra bites, 23, 16, and 15 patients received debridement, fasciotomy, and graft.

### 2.2. Wound Conditions of the Tissue Necrosis Group

Of the 23 patients, 14 had positive wound culture findings (positive culture rate, 60.87%), and among them, eight patients received all the following surgical procedures: debridement, fasciotomy, and graft. Of these 14 patients, 3, 2, and 1 accepted debridement/graft, debridement, and debridement/fasciotomy. Swab cultures for both aerobic and anaerobic bacteria were taken during debridement/fasciotomy procedures. If prominent pus or wound discharge was noted, swab cultures were collected and analyzed for both aerobic and anaerobic bacteria. Of these 14 patients with wound necrosis, six and eight had monomicrobial and polymicrobial infections. The most commonly cultured aerobic Gram-negative and Gram-positive bacteria were *Morganella morganii* and *Enterococcus faecalis*, and both were found in 11 of the 14 patients. Coagulase-negative *staphylococcus* was the third leading pathogen in this study. However, anaerobic pathogens were identified in the cultures of only six patients (four and two in the necrosis and nonnecrosis groups).

Notably, wound cultures of some patients were obtained several times, even up to seven times, during their hospitalization course. *E. faecalis* and *M. morganii* were the two most commonly obtained bacteria in first-time wound cultures. Moreover, anaerobic bacteria such as *Bacteroides fragilis*, *Providencia rettgeri*, *Proteus vulgaris*, and *Serratia marcescens* were observed in patients whose wound cultures were obtained multiple times when they underwent several surgical procedures (Table 1). The results of the antibiotic sensitivity tests are included in Table S1.

### 2.3. Wound Conditions of the Nonnecrosis Group

In contrast to the wound necrosis group, only six patients had positive wound cultures in non-necrosis group (*n* = 172), with three each having monomicrobial and polymicrobial infections. Five cultures showed *M. morganii* growth and two showed *E. faecalis* growth (Table 2). Among these six patients, only one (Case 6) received fasciotomy and graft due to of clinically diagnosed compartment syndrome.

### 2.4. Development of the Clinical Prediction Rule for Wound Infections (Cobra BITE Score)

Among the 195 patients with cobra bites, 23 with tissue necrosis who required antibiotics for wound management were not included in the analysis of Cobra BITE score. In total, 172 patients without tissue necrosis were enrolled and divided into wound infection and no wound infection groups. Men accounted for the majority of the patients in both groups, but no statistical differences were observed in both age and sex. The wound infection group had higher white blood cell (WBC) counts, neutrophil counts (band and segment), and neutrophil-lymphocyte ratios (NLRs), but no statistical differences were observed among them, except in NLR (*p* = 0.04). The wound infection group had high levels of myoglobin and blood glucose (*p* = 0.03 for both); however, no statistical differences were noted in the following clinical characteristics: platelet count, prothrombin time (PT), activated partial thromboplastin time (APTT), blood urea nitrogen (BUN), creatinine (Cr), sodium, and potassium. In terms of treatment and outcome, patients in the wound infection group received more antivenin (*p* = 0.02), had a higher admission rate (*p* < 0.0001), and had a longer hospitalization time (*p* = 0.01) than those in the non-infection group (Table 3).

**Figure 2:**
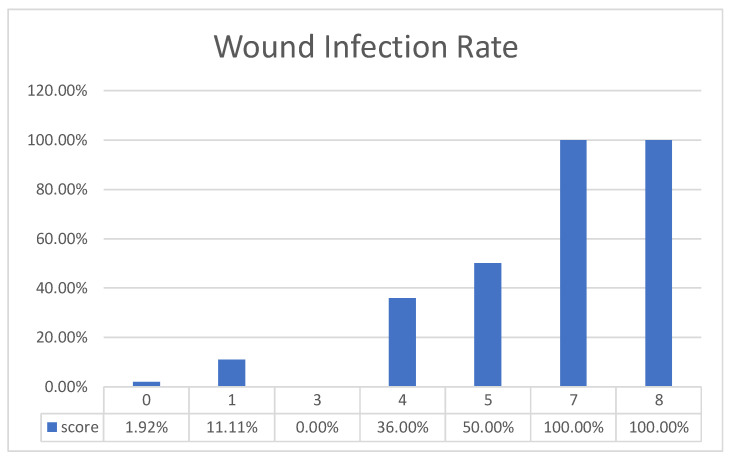
Cobra BITE scores and wound infection rates. Cobra BITE scores and wound infection rates. The higher the score was, the higher the wound infection rate. With an optimal cutoff point of 7, the sensitivity and specificity of Cobra BITE score were 0.89 and 0.76, respectively.

**Figure 3:**
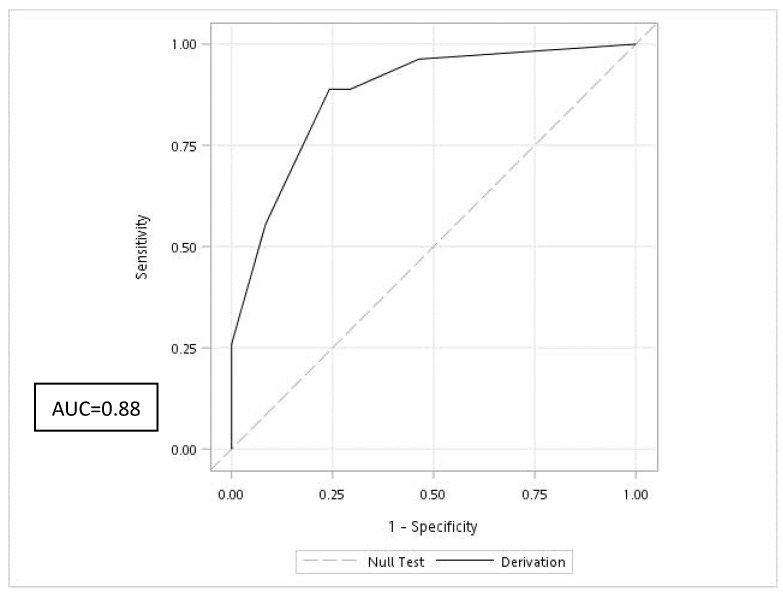
Receiver operator characteristic curve of Cobra BITE score. AUC: area under the curve. The receiver operator characteristic curve of Cobra BITE score was 0.88.

Clinical characteristics with a *p* value of <0.1 were further analyzed through multiple logistic regression to identify independent predictors of wound infections, namely a WBC (in 10^3^/µL) × NLR value of ≥114.23, antivenin dose, and hospital admission (Table 4). The β coefficient of each variable was divided by 0.4 and rounded off to the nearest integer to form the prediction rule. The stratification of our study population according to the Cobra BITE score showed rising infection rates (Figure 2). The area under the receiver operating characteristic (ROC) curve of the prediction rule was 0.88 (Figure 3). With an optimal cutoff point of 7, the prediction rule was ideal at predicting 100% of wound infections, with sensitivity and specificity of 0.89 and 0.76 and positive and negative predictive values of 0.51 and 0.96, respectively. The Hosmer-Lemeshow *p* value was 0.4.

## 3. Discussion

In this study, we had the cobra bites incidence of 21.03%, which was consistent with previous studies [1,7,8]. To our knowledge, our study is the largest multicenter study to examine the bacteriology and prediction factors of cobra bite wounds. The infection rate of Taiwan cobra bites varied has varied from 28% [3] to 68% [4] and was as high as 77% [5] in different studies. We found that 27.17% of our patients with cobra bites had wound infections; this rate was low compared with those in previous studies. One of the reasons for the discrepancy in infection rates may be the different geographic distribution of the Taiwan cobra. The highest cobra bite wound infection rate was in central Taiwan, which had the highest cobra bite incidence [1]. The second reason may be that the study hospital is the main center in central Taiwan to which patients with snakebites are referred, and therefore, patients with severe cobra envenomation were transferred there, giving a falsely high infection rate. In our study, we found that patients with severe envenomation had wound infection.

### 3.1. Bacteriology, Culture Times, and Choice of Antibiotics for Patients with Taiwan Cobra Bites

Up to 14 different pathogens have been identified in this study. All of these isolated pathogens were skin commensals or opportunistic pathogens. When comparing pathogens isolated from necrosis and nonnecrosis wounds, we found that patients of the necrosis wound group were more easily to be prone to have polymicrobial and more complicated wound infection. We believed that patients with wound necrosis who required multiple surgical interventions were at risk of developing their complicated infections.

There are two factors we should consider in exploring the bacteriology of infected snakebites. First, the bacteriology of infected snakebite wounds reflects the polymicrobial flora of snakes’ oral cavities because of the direct inoculation of the pathogens from the snake’s oral cavity to the wound. For example, *Morganella morganii*, *Aeromonas hydrophilia,* and *E. faecalis* are frequently isolated from the oral cavity of snakes [9,10,11,12,13,14]. It is thus not surprising that they are some of the most common pathogens found in infected snakebite wounds [3,4,6,9]. Second, venom induced tissue damage allowing for the opportunistic colonization of the wound by snake oral cavity flora, skin commensals, or environmental bacteria. This was demonstrated in a study regarding wound infection in Taiwan habu/ green bamboo viper snakebites patients [15]. Either Taiwan habu (*Protobothrops mucrosquamatus*) or green bamboo viper (*Viridovipera stejnegeri*) have their venom rich in metalloproteinases and phospholipase A2, which have cytotoxic, myotoxic, and pro-inflammatory properties and thus cause tissue damage [16,17]. Both in the previous study [15] and this study, we found that patients with infected snakebite wounds were administered more doses of antivenin compared to those without infected wounds. The more venom that is injected, the more severe the resultant tissue damage and consequent infection. It is accepted that more cytotoxin dose may induce tissue necrosis to a more extensive degree of tissue necrosis [18]. In this study, all of the patients with tissue necrosis were highly infected and had more complicated bacteriology than the nonnecrosis wound infection patients. Thus, the degree of tissue destruction might be one of the contributing factors causing wound infection.

Considering the bacterial culture of wound infections, irrespective of whether the patients had wound tissue necrosis, snakebite wound infection is usually polymicrobial, with *M. morganii* and *E. faecalis* being the most often identified pathogens [3,5,6,9]. Therefore, using gentamicin, ceftriaxone, ciprofloxacin, or levofloxacin as the first-line monotherapy drug is reasonable for managing *N. atra* bite wound infections. Since *N. atra* bites cause considerable wound tissue swelling or necrosis [19,20] that may lead patients to receive multiple surgical procedures, using antibiotics such as metronidazole, augmentin, or piperacillin/tazobactam to cover both aerobic and anaerobic microorganisms was recommended [21]. However, unlike previous studies, *Pseudomonas aeruginosa* was not observed in this study [3,5,6]. This finding suggested that the choice of antibiotics used should be based on local bacterial patterns according to drug susceptibility tests. Therefore, ureidopenicillins such as piperacillin should be reserved for patients prone to *P. aeruginosa* infection.

*E. faecalis* was one of the most isolated pathogens found in this study. We observed that antibiotic resistance existed in some of the bacteria cultures, thus reminding us of the occurrence of vancomycin-resistant *Enterococci* (VRE) in the cobra bite wounds. Therefore, sticking strictly to using antibiotics according to the above suggestions of choice of antibiotics according to local bacterial pattern and their drug susceptibility tests is of paramount importance in preventing the occurrence of VRE. Once VRE is cultured, the choice of antibiotics would be linezolid, daptomycin, or tigecycline [22,23].

Other measurements, such as washing hands, remove the possible VRE colonization site (such as the necrotic tissue, central lines, or removing the unnecessary Foley catheter) and should also be implanted to prevent spread within the hospital.

In conclusion, we recommended the stepwise use of antibiotics and the employment of appropriate surgical interventions, such as debridement, to treat wound infections from Taiwan cobra bites successfully.

### 3.2. Factors Related to Cobra BITE Score and Its Association with a Secondary Bacterial Infection from Taiwan Cobra Bites

Although moderate to high superimposed bacterial infections have been observed in patients with Taiwan cobra bites, no clinical rules have been compiled to acknowledge this. Some studies have suggested that snakebite wound infections should be considered a special infection type that requires objective measurements [5,21]. Our Cobra BITE score was conceived for this purpose.

The Cobra BITE score has three components: WBC × NLR, hospital admission, and antivenin dose. Generally, these three items are associated with severity degree, as suggested by other studies, and moderate severe patients or patients with severe snakebite envenomation often experience wound infections [24,25,26]. Using this score is convenient, because WBC, NLR, hospital admission data, and antivenin dose can be determined in the early stage of snakebite treatment. Thus, appropriate antibiotics can be administrated to suitable patients.

In this study, hospital admission was the best predictor of wound infections. Doctors admit patients with severe bites (such as those who received a high antivenin dose) to hospital for further care, because patients with moderate or severe snakebites often develop wound infections. Furthermore, wound infections were found to occur mostly in moderate or severe snakebite cases [24,25,26]. Therefore, secondary wound infections in patients with snakebites are sufficiently serious to warrant hospital admission.

Both WBC and NLR are biomarkers routinely used to determine infection. Several diseases and clinical conditions, such as trauma, infection, sepsis, operation, malignancy, and emotional instability, can cause leukocytosis. Generally, any factor that results in stress can induce leukocytosis [27]. Moreover, a study showed WBC elevation to be a risk factor for post-snakebite compartment syndrome [28]. Conversely, compared with leukocytosis, NLR increase can be detected earlier (when patients have a proinflammatory state), and this can make clinicians aware of potential infections and systemic inflammation [29]. In experimental studies using dogs, neutrophil concentration increased and a left shift occurred between 4 and 12 h after bacterial inoculation [30]. As patients with snakebites often seek medical help within 4 h in Taiwan, using NLR to predict wound infection is reasonable. Some studies have indicated that an NLR increase is related to vascular endothelium injury, which can result in poor wound healing [31]. NLR has been used to evaluate diabetes mellitus-related foot infections [32], vascular ischemia of the limbs [33], and sepsis [34,35]. Therefore, we integrated WBC and NLR to evaluate wound healing and inflammation precisely. If additional studies can confirm that some other local/circulatory inflammatory mediators or venom concentration are sensitive and precise for predicting wound infections, such inflammatory mediators can be incorporated into the Cobra BITE score to further improve its utility at predicting wound infections in patients with snakebites.

### 3.3. Utility of the Cobra BITE Score

Our score system has some advantages for clinical decision making. First, its primary use is to enable the judicious use of antibiotics in patients with Taiwan cobra bites without wound tissue necrosis. According to local bacteriological data, antibiotic use can be suitable for patients with cobra bite wound infections with and without tissue necrosis. Second, these decision-making processes can be extended to patients bitten by other *Naja* snakes. *Naja* bites are major contributors to the snakebite burden globally [36]. Bites from different *Naja* species share common features of local tissue swelling, inflammation/infection, and substantial tissue necrosis. These *Naja* species are *N. atra* (Taiwan cobra) [37], *Naja siamensis* (Thai spitting cobra) [38], *Naja kaouthia* (monocellate cobra) [39,40], *Naja naja* (Indian cobra) [41,42], and Mozambique spitting cobra (*Naja mossambica*) [43,44]. Our Cobra BITE score offers the medical community a framework for stratifying the risk of wound infection after cobra bites, and it can be improved upon and refined further. Future studies on the aforementioned *Naja* species can be conducted to develop individual Cobra BITE scores, enabling the judicious use of antibiotics in patients with cobra bites. Furthermore, studies can be conducted to investigate the use of antibiotic prophylaxis in patients with cobra bites at a high risk of wound infections.

### 3.4. Differences between Naja atra Bites Wound Infections and Protobothrops mucrosquamatus and Viridovipera stejnegeri Bites Wound Infection in Taiwan

As we described in the introduction section, there are six clinically medically important venomous snakes in Taiwan. *Naja atra*, *Protobothrops mucrosquamatus*, and *Viridovipera stejnegeri* accounted for more than 95% of snakebites. In general, *Naja atra* bites had much higher wound infection than *Protobothrops mucrosquamatus* and *Viridovipera stejnegeri* associated wound infections. Only seven cases in 163 victims who suffered from wound infection showed positive culture findings in our previous report [15]. However, the cultured microorganism species were similar. *Enterococcus faecalis* and *Morganella morganii* were still the most commonly found pathogens.

### 3.5. The Role of Antivenom in Preventing the Development of Cobra Bites Wound Infection

Antivenom is the fundamental treatment of cobra snakebites. Most of the cobra bites patients receive antivenom for the consequence of cobra bites of neurological or local limbs swelling. Antivenom itself is sufficient to treat the least severe cobra-envenomated patients, and there are no further complications. In our study, all the nonnecrosis group patients received antivenom, and only a few of them had wound infection. On the other hand, whether antivenom can prevent the development of tissue necrosis is still in debate. Since we think all the necrotic wounds are infected, antivenom retains its role in neutralizing the venom but has no position in preventing wound infection in such kinds of patients.

### 3.6. Limitations

Our study has some limitations. This was a retrospective study, and the study design has inherent limitations (such as recall bias). We attempted to overcome these innate limitations through systematically retrieving data from the CGRD, which is based on original electronic medical records. However, the results should be interpreted with caution. The second limitation of this study is that the Cobra BITE score has not yet been validated. Nevertheless, the Hosmer-Lemeshow analysis of our Cobra BITE score supports the model’s stability.

## 4. Conclusions

We offer a treatment and study framework for cobra bite wound infections. When treating a cobra bites patient, we should assess the biting site if there is tissue necrosis, order hematological parameters such as WBC/NLR ratio in addition to antibiogram, and choose the antibiotic properly. According to our study results, we recommend using antibiotics in a stepwise manner for all patients with infected wounds. Gentamicin, ceftriaxone, ciprofloxacin, and levofloxacin are reasonable first-line monotherapies. If patients receive multiple surgical procedures, the use of metronidazole, augmentin, and piperacillin/tazobactam is encouraged to cope with the possible anaerobic wound infection. However, ureidopenicillin should be reserved for patients at risk of *Pseudomonas* spp. infection. For those patients infected with VRE, the choice of antibiotics would be linezolid, daptomycin, or tigecycline. Washing hands and removing the possible VRE colonization site (such as the necrotic tissue, central lines, or removing the unnecessary Foley catheter) should also be implanted to prevent its spread within the hospital.

Last, doctors could employ our Cobra BITE score in further studies regarding the *Naja* spp.-associated wound infections.

## 5. Materials and Methods

### 5.1. Ethics Statement

To meet research ethics standards, this study was conducted after permission was received from the Chang Gung Memorial Hospital (CGMH) Research Ethical Committee, Taoyuan, Taiwan (Approval No: 201800736B0, date of approval: 21 May 2018). The requirement of consent from study participants was waived in accordance with relevant guidelines and regulations.

### 5.2. Data Resource and Setting

The CGMH network, a private hospital network of seven hospitals in Taiwan, established in 1976, is located in the northeastern and southern regions of Taiwan. The CGMH network is the largest medical network in Taiwan, and this medical group has approximately 10,070 beds and handles >280,000 admissions every year. Thus, 10.2% of patients admitted to a hospital in Taiwan each year attend a CGMH branch. Outpatient visits and ED visits to CGMH branches are approximately 8,500,000 and 500,000 per year [45].

The variables analyzed in this study were retrieved from the CGRD, a computerized deidentified database, which is systematically updated to include new data generated annually. The CGRD was derived from original CGMH medical records. Its overall coverage of 21.2% and 12.4% of outpatient and inpatient records, respectively, means its data can provide a good foundation for high-quality and scientifically sound studies [46].

### 5.3. Patients Enrolled

All patient with Taiwan cobra bites who presented to the ED of a CGMH and received at least one vial of freeze-dried neurotoxic antivenin (the designated antivenin for Taiwan cobra bites) between January 2001 and May 2017 were identified using the International Statistical Classification of Diseases, ninth and 10th revision codes for diagnosing snakebites or determining antivenin administration. Patients receiving any other antivenin medication were excluded.

The following variables were collected: demographic characteristics (patient age and sex); laboratory variables such as complete blood count with differential count, NLR, hemoglobin, red blood cell distribution width, platelet count, PT, APTT, BUN, Cr, alanine aminotransferase, aspartate aminotransferase, myoglobin, potassium, sodium, and blood glucose. Data on the bacteriology of wounds and pus culture, such as antimicrobial drug susceptibility of microorganisms obtained from swabs of snakebite wounds, were also retrieved. Information on treatment modalities such as antivenin doses, type of surgical procedure (debridement, fasciotomy, or graft), hospital admission, and hospitalization length was retrieved. Based on a previous study, the term polymicrobial infection was used to describe the growth of two or more microorganisms on the same infected wound [47].

### 5.4. Management Protocol for Patients with Venomous Snakebite

The management protocol for patients with venomous snakebites was adequately described in our previous work [15]. A short-term study revealed that all patients who presented with a snakebite to our EDs were managed in accordance with World Health Organization guidelines [48]. After the culprit snake was identified (through a pictorial chart available in our EDs and compatible symptoms and signs), antivenin was appropriately administrated to patients. Patients were then monitored in the ED for 24 h post-bite until clinical improvement was observed in limb pain and swelling, after which those who were hemodynamically stable were allowed to go home. If limb swelling reappears after adequate antivenin administration, or if clinical signs of cellulitis were observed, these patients were diagnosed as having a wound infection and were admitted to our wards. Patients with wound necrosis may receive surgical procedures such as debridement, fasciotomy, or tissue graft after thorough clinical consideration. This protocol remained consistent throughout our study period.

### 5.5. Definitions of Wound Infection and Wound Necrosis

Patients were considered to have infected snakebite wounds if they satisfied one of the following criteria: (1) had positive wound cultures; (2) had admission diagnoses of cellulitis, abscess, or necrotizing fasciitis; or (3) underwent surgical wound debridement. We defined patients who received debridement as those who had wound necrosis because such patients require this procedure.

### 5.6. Statistical Analysis and Development of the Cobra BITE Score

Categorical variables are reported as frequencies and percentages, whereas continuous variables are expressed as mean ± standard deviation, unless otherwise indicated. For univariate analyses, we conducted Student’s *t*-test and a chi-squared test to evaluate numerical and categorical variables separately. To assess the strength of the association between two groups and express statistical differences, odds ratios and 95% confidence intervals were used. Variables with *p* < 0.1 were identified as possible predictors for further multivariate analysis using multiple logistic regression. We used Youden’s index or the most optimal value(s) to determine the cut-off points for Cobra BITE score variables. Through weighting these variables according to their β coefficients, the Cobra BITE score was then created. A Hosmer-Lemeshow analysis was used to test the model’s stability. Finally, the ROC curves and area under the curve were used to determine the accuracy of this prediction model. A *p* value of <0.05 was considered statistically significant. All statistical analyses were performed using SAS statistical software version 9.2 (SAS Institute, Cary, NC, USA, 2013).

## Figures and Tables

**Table 1:** Microorganisms found in the wound bacterial culture of the necrosis group.

No.	Culture Sequence ^@^	Microorganism		
		Aerobic Gram-Positive	Aerobic Gram-Negative	Anaerobic
1	1	*Enterococcus faecalis*	*Morganella morganii*	
2	1/2	*Enterococcus faecalis*	*Morganella morganii*	
3	1		*Morganella morganii*	
4	1/2 *	*Enterococcus faecalis*	*Morganella morganii*	
5	2/1	*Enterococcus faecalis,* *Viridans streptococcus*	*Morganella morganii*	
	4/3	*Enterococcus faecalis*		*B.fragilis*
	6/5	Gm(+) bacilli		*Providencia rettgeri*
	7	*Enterococcus faecalis*		
6	2/1	*Enterococcus faecalis*	*Morganella morganii*	
	3/4	*Enterococcus faecalis*	*Morganella morganii*	
7	1		*Morganella morganii*	
8	1	*Enterococcus faecalis*		
	2/3/4	*Enterococcus faecalis,* *Coagulase negative staphylococcus*		
9	1&2	*Enterococcus faecalis*	*Morganella morganii*	
10	1	*Enterococcus faecalis*	*Morganella morganii*	*Proteus vulgaris*
	2	*Enterococcus faecalis, Coagulase negative staphylococcus*	*Morganella morganii*	
	4/3		*Acinetobacter sp.*	*B.thetaiotaomicron*
11	1	*Enterococcus faecalis*		
	2/3	*Enterococcus faecalis*	*Morganella morganii*	
12	1	*Enterococcus faecalis*	*Morganella morganii*	
	2	*Bacillus,* *Coagulase negative staphylococcus*	*Acineto.baumannii*	
	3	*Coagulase negative staphylococcus,*	*Acineto.baumannii*	
13	1&3	*Enterococcus faecalis*		*Serratia marcescens*
	4/2	*Enterococcus faecalis*		*B.fragilis*
14	2/1	*Staph.aureus*	*Shewanella algae*	

@ culture sequence, 1/2= culture results of 1st and 2nd time culture; 1&2 =1st and 2nd time culture had the same culture result; * Yeast-like organism also cultured.

**Table 2:** Microorganisms found in the wound bacterial culture of the nonnecrosis group.

No.	Culture Sequence ^@^	Microorganism		
		Aerobic Gram-Positive	Aerobic Gram-Negative	Anaerobic
1	1		*Morganella morganii*	
2	1	*Enterococcus faecalis*		
	2	*Enterococcus faecalis*	*Morganella morganii*	
3	1			*Citrob.freundii*
4	1	*Enterococcus faecalis*	*Morganella morganii*	
5	1		*Morganella morganii*	
6	1		*Shewanella algae*	
	2		*Morganella morganii*	*Serratia marcescens*
	3	*Growth for aerobes*		

@ culture sequence same as Table 1.

**Table 3:** Patient demographics, laboratory results, and treatment modalities used in the univariate analysis.

Variable	Patients		*p-*Value
	No Wound Infection *n* = 142	Wound Infection *n* = 30	
Demographic characteristics			
Age, Mean (SD)	45.54 (17.18)	50.83 (18.74)	0.51
Male, n (%)	101 (71.13)	24 (80.00)	0.26
Laboratory variables			
WBC(1000/μL), Mean (SD)	8.19 (3.12)	9.18 (4.27)	0.27
Band, Mean (SD)	0.05 (0.40)	0.13 (0.51)	0.34
Segment, Mean (SD)	61.72 (13.91)	67.56 (19.81)	0.16
Lymphocyte, Mean (SD)	30.08 (12.00)	25.90 (18.95)	0.25
Neutrophile lymphocyte ratio	3.31 (4.16)	7.27 (9.11)	0.04
WBC × NLR, Mean (SD)	35.62 (72.74)	93.29 (144.4)	0.05
HB (g/dL), Mean (SD)	13.91 (1.74)	14.24 (1.69)	0.39
RDW, Mean (SD)	16.11 (9.65)	15.19 (7.64)	0.65
PLT (1000/μL), Mean (SD)	212.4 (53.08)	210.8 (48.22)	0.89
Prothrombin time, Mean (SD)	11.23 (1.77)	10.94 (0.92)	0.27
aPTT, Mean (SD)	28.53 (8.51)	26.62 (3.00)	0.09
Cr (mg/dL), Mean (SD)	1.15 (1.50)	1.10 (1.18)	0.87
BUN (mg/dL), Mean (SD)	15.89 (12.33)	12.55 (4.87)	0.13
ALT/GPT (U/L), Mean (SD)	26.61 (12.73)	30.90 (32.32)	0.48
AST /GOT, Mean (SD)	42.76 (47.49)	42.33 (26.70)	0.97
Creatine_kinase, Mean (SD)	154.1 (55.84)	372.0 (426.6)	0.17
myoglobin, Mean (SD)	88.11 (106.5)	36.43 (22.26)	0.03
K (mEq/L), Mean (SD)	3.62 (0.38)	3.75 (0.32)	0.17
Na (mEq/L), Mean (SD)	139.5 (2.02)	139.6 (2.33)	0.92
Glu (mg/ dL), Mean (SD)	121.7 (30.14)	141.3 (39.39)	0.03
Treatment modalities			
Antivenin, vial, Median(IQR)	2 (3–1)	2.5 (6–1)	0.02
Hospitalization			
hospital admission, n (%)	27 (19.01)	24 (80.00)	<0.0001
length of hospitalization, Mean (SD)	3.07 (2.02)	6.46 (4.01)	0.01

WBC × NLR, WBC multiplied by NLR.

**Table 4:** Multiple logistic regression and wound infection prediction rule.

Variable	*β* ^※^	Odds Ratio	95% Confidence Interval	Points
Intercept	−0.6935			
WBC × NLR (≥114.23)	1.0550	8.249	(1.452, 46.853)	3
Admission	1.6161	25.336	(6.408, 100.177)	4
Antivenin dose (≥4)	0.4018	2.234	(0.735, 6.788)	1

^※^*β*: vector of weights (or regression coefficients) corresponding to outcome. The *β* coefficient of each variable was divided by 0.4 and rounded off to the nearest integer to form the prediction rule.

## Data Availability

Data sharing not applicable.

## Supplementary Materials

The following are available online at https://www.mdpi.com/2072-6651/13/3/183/s1, Table S1: Results of the antibiotic sensitivity tests.
